# The incidence and outcome of AKI in patients with sepsis in the emergency department applying different definitions of AKI and sepsis

**DOI:** 10.1007/s11255-022-03267-5

**Published:** 2022-07-20

**Authors:** Maarten Cobussen, Jacobien C. Verhave, Jacqueline Buijs, Patricia M. Stassen

**Affiliations:** 1 Department of Internal Medicine, Division of General Medicine, Section Acute Medicine, Maastricht University Medical Center, Maastricht, The Netherlands; 2 Department of Internal Medicine, Rijnstate Hospital, Arnhem, The Netherlands; 3 Department of Internal Medicine, Zuyderland Medical Center, Heerlen, The Netherlands; 4 CAPHRI School for Public Health and Primary Care, Faculty of Health, Medicine and Life Sciences, Maastricht University, Maastricht, The Netherlands

**Keywords:** Acute kidney injury, AKI, RIFLE, AKIN, AKIB, Delta check, KDIGO, Sepsis, SIRS, qSOFA, SOFA, Emergency department

## Abstract

**Background:**

Sepsis is often accompanied with acute kidney injury (AKI). The incidence of AKI in patients visiting the emergency department (ED) with sepsis according to the new SOFA criteria is not exactly known, because the definition of sepsis has changed and many definitions of AKI exist. Given the important consequences of early recognition of AKI in sepsis, our aim was to assess the epidemiology of sepsis-associated AKI using different AKI definitions (RIFLE, AKIN, AKIB, delta check, and KDIGO) for the different sepsis classifications (SIRS, qSOFA, and SOFA).

**Methods:**

We retrospectively enrolled patients with sepsis in the ED in three hospitals and applied different AKI definitions to determine the incidence of sepsis-associated AKI. In addition, the association between the different AKI definitions and persistent kidney injury, hospital length of stay, and 30-day mortality were evaluated.

**Results:**

In total, 2065 patients were included. The incidence of AKI was 17.7–51.1%, depending on sepsis and AKI definition. The highest incidence of AKI was found in qSOFA patients when the AKIN and KDIGO definitions were applied (51.1%). Applying the AKIN and KDIGO definitions in patients with sepsis according to the SOFA criteria, AKI was present in 37.3% of patients, and using the SIRS criteria, AKI was present in 25.4% of patients. Crude 30-day mortality, prolonged length of stay, and persistent kidney injury were comparable for patients diagnosed with AKI, regardless of the definition used.

**Conclusion:**

The incidence of AKI in patients with sepsis is highly dependent on how patients with sepsis are categorised and how AKI is defined. When AKI (any definition) was already present at the ED, 30-day mortality was high (22.2%). The diagnosis of AKI in sepsis can be considered as a sign of severe disease and helps to identify patients at high risk of adverse outcome at an early stage.

## Introduction

Acute kidney injury (AKI) is a common and serious complication of sepsis. It is caused by several factors, including a dysregulated host response to an infection, which eventually leads to organ failure [1]. The incidence of AKI in sepsis ranges widely from 15 to 87%, depending on which population is studied and how AKI and sepsis are defined [2, 3].

Early recognition of AKI in patients with sepsis is important, because it is associated with increased mortality and morbidity. In addition, close monitoring of the complications of renal failure are required to preserve and improve renal function [4, 5]. In international literature, a variety of definitions for AKI are described. Most commonly used are the RIFLE (Risk, Injury, Failure, Loss of kidney function, and End-stage kidney disease), AKIN (Acute Kidney Injury Network), and KDIGO (Kidney Disease: Improving Global Outcomes). Other definitions used are AKIB and the delta check (Table 1) [3, 6]. Moreover, in 2016, a new definition for sepsis was introduced replacing the SIRS criteria (Sepsis-2) with the qSOFA and SOFA criteria (Sepsis-3) [7]. Variations in both classification criteria for sepsis as well as AKI make the interpretation of previous studies on the incidence of AKI in sepsis difficult.

**Table 1:** Definitions for AKI

	RIFLE	AKIN	AKIB	Delta check	KDIGO
Staging	Serum creatinine	Serum creatinine	Serum creatinine	Serum creatinine	Serum creatinine
1 (Risk)	> 1.5 × baseline	> 1.5 × baseline OR ≥ 26.5 increase	> 26 increase over 24 h OR > 44 over 48 h	> 26 increase	> 1.5 × baseline OR ≥ 26.5 increase
2 (Injury)	> 2 × baseline	> 2 × baseline	> 44 increase over 24 h OR > 88 over 48 h		> 2 × baseline
3 (Failure)	> 3 × baseline OR ≥ 44 increase if ≥ 300	> 3 × baseline OR ≥ 44 increase if baseline ≥ 353 OR requiring RRT	> 88 increase over 24 h OR > 132 over 48 h		> 3 × baseline OR ≥ 353 OR requiring RRT

Serum creatinine is in μmol/L

Acute Dialysis Quality Initiative Group RIFLE: Risk, Injury, Failure, Loss, End Stage Renal Disease [12]; AKIN: Acute Kidney Injury Network [13]; AKIB: Acute Kidney Injury Bonventre [14]; delta check [15]; KDIGO: Kidney Disease Improving Global Outcomes [16]; RRT: renal replacement therapy

RIFLE categories loss (requiring > 4 weeks of RRT) and end-stage renal disease (requiring > 12 weeks of RRT) were not taken into account, because these groups of patients were not included in the study and therefore withdrawn from the table

Next to this, previous studies on the incidence of sepsis-associated AKI almost exclusively focussed on ICU patients. Only a few studies described patients presenting with sepsis in the emergency department (ED). However, these studies used older definitions for both AKI and sepsis [3, 6]. The incidence of AKI in patients with sepsis according to the new SOFA criteria presenting at the ED is therefore uncertain.

Given the important clinical consequences of early recognition of AKI in sepsis, the aim of this multicentre retrospective study was to investigate the incidence of AKI in patients with sepsis at presentation at the ED, according to five AKI classifications (RIFLE, AKIN, AKIB, delta check, and KDIGO) and three sepsis criteria (SIRS, qSOFA, and SOFA). The KDIGO criteria for AKI and the SOFA criteria for sepsis were used as reference, since these are the newest and most widely accepted.

## Methods

### Study design

We performed a retrospective study in three large teaching hospitals in the Netherlands (Maastricht University Medical Centre (MUMC), Rijnstate Hospital, and Zuyderland Medical Centre). Patients were enrolled between January 2015 and December 2016 (MUMC) and between July and December 2015 (Rijnstate Hospital and Zuyderland Medical Centre).

### Study population

All patients ≥ 18 years of age who were treated at the ED and consecutively admitted to the internal medicine department and who fulfilled the sepsis criteria—either SIRS, qSOFA, or SOFA—were included. Patients were excluded when they had a post-renal cause of AKI established within 48 h of admission, when they already received concomitant chronic renal replacement therapy (RRT), or when no creatinine values were available at the time of admission.

### Data collection

All data were retrieved from the electronic hospital charts. Standardized scoring forms were used to extract age, sex, weight, length, vital parameters, SIRS, qSOFA, and SOFA criteria [7]. According to the SOFA criteria, sepsis was defined when a patient met ≥ 2 criteria for organ failure. Shock was defined as sepsis accompanied by a lactate > 2 mmol/l and the need of vasopressors to maintain an MAP ≥ 65 mmHg despite adequate fluid resuscitation [7]. At presentation at the ED, the pre-existing SOFA score was assumed to be zero [8]. Prior medical history, the Charlson Comorbidity Index [9], risk factors for development of AKI, such as diseases potentially influencing renal function, and the use of drugs interfering with renal function were retrieved. Also, 30-day all-cause mortality was documented.

### Renal function and outcome measures

The primary outcome for analysis was the incidence of AKI in patients with sepsis, according to the SIRS, qSOFA, and SOFA criteria, using the SOFA criteria as reference. AKI was defined by the RIFLE, AKIN, AKIB, delta check, and KDIGO criteria, using the KDIGO criteria as reference.

To establish baseline renal function (renal function prior to the sepsis episode), creatinine values up to 3 months prior to ED presentation were obtained [10]. When these were not available, we used the lowest creatinine value during 3 month follow-up as baseline creatinine [11]. Baseline eGFR was calculated using the CKD-EPI (Chronic Kidney Disease Epidemiology Collaboration) formula. Chronic kidney disease was defined as a baseline serum creatinine > 178 µmol/l (as part of the Charlson Comorbidity Index).

AKI at the time of presentation at the ED was classified according to the RIFLE, AKIN, AKIB, delta check, and KDIGO criteria for AKI (Table 1) [12–16]. Urine production was not monitored nor documented in a standardized way and was therefore withdrawn from all AKI scores, like in other studies on AKI in the setting of an ED [17, 18].

In addition, we studied the association between the secondary outcome measures: persistent kidney injury, hospital length of stay, 30-day mortality, and AKI at admission, as defined by the KDIGO criteria. Persistent kidney injury was defined as at least a stage 1 AKI or any higher AKI classification during the second week after admission compared to baseline renal function (renal function prior to the sepsis episode).

### Analysis and statistics

Statistical analysis was performed using IBM SPSS version 22 (SPSS Inc., USA). Continuous variables were reported as median [interquartile range (IQR)], and categorical variables as proportions. Comparisons between two groups were made using Mann–Whitney test for continuous data and Pearson’s chi-squared test for categorical data. Comparisons between multiple groups were made using Kruskal–Wallis test for continuous and Fisher’s exact test for categorical data. Hereafter, when a significant difference was found, a post hoc analysis was used to test for differences between the groups. For the post hoc analysis, the Bonferroni adjustment was used.

Logistic regression analysis was used to assess the association of each AKI severity with 30-day mortality without correction for other confounders. Data are presented as odds ratios (OR) with 95% confidence intervals (CI). The overlap between the different AKI definitions were identified and visualized by a Venn diagram, plotted using jvenn (source: http://jvenn.toulouse.inra.fr/app/index.html) [19]. P values < 0.05 were considered statistically significant.

### Ethical approval

The Ethics Committees of all participating hospitals approved this study with a waiver of informed consent (METC 13-4-103.12).

## Results

During the study period, a total of 2065 patients fulfilled at least one sepsis definition. Of these patients, 2011 (97.4%) met the SIRS criteria, 315 (15.3%) patients met the qSOFA criteria, and 1246 (60.3%) fulfilled the SOFA criteria for sepsis (Table 2).

**Table 2:** Characteristics of patients stratified by different sepsis definitions

	SIRS, *n* = 2011	qSOFA, *n* = 315	SOFA, *n* = 1246
Age (years)	69 (56–79)	77 (65–83)	72 (61–81)
Male sex	1049 (52.2)	159 (50.5)	708 (56.8)
BMI	25.1 (22.1–29.1)	25.0 (21.8–29.1)	25.2 (22.1–29.1)
Age-adjusted CCI	6 (4–7)	6 (5–8)	6 (4–7)
Diabetes mellitus	155 (7.7)	34 (10.8)	112 (9.0)
Liver disease	44 (2.2)	11 (3.5)	39 (3.1)
Chronic kidney disease	171 (8.5)	36 (11.4)	145 (11.6)
Baseline creatinine (μmol/L)	81 (66–107)	84 (66–116)	89 (69–125)
Baseline eGFR (CKD-EPI) (ml/min/1.73 m^2^)	75 (53–93)	66 (49–89)	67 (44–88)
Baseline urea (mmol/L)	6.7 (4.8–10.1)	8.6 (6.2–14.8)	8.2 (5.6–12.8)
NSAID	98 (4.9)	11 (3.5)	53 (4.3)
ACEi/ARB	616 (30.6)	117 (37.1)	425 (34.1)
Diuretics	592 (29.4)	128 (40.6)	438 (35.1)
Shock	136 (6.8)	n.a.*	63 (5.1)
ICU admission	134 (6.7)	75 (23.8)	121 (9.7)

Data are presented in medians (interquartile ranges) or in absolute numbers (percentages)

*SIRS* systemic inflammatory response syndrome, *qSOFA* quick sequential organ failure assessment, *SOFA* sequential organ failure assessment, *BMI* body mass index, *eGFR* estimated glomerular filtration rate, *CKD-EPI* Chronic Kidney Disease Epidemiology Collaboration, *CCI* Charlson Comorbidity Index, *NSAID* non-steroidal anti-inflammatory drug, *ACEi* ace inhibitor, *ARB* angiotensin receptor blocker

*The qSOFA criteria only define sepsis and not shock

### Acute kidney injury by sepsis definition

Of the patients with sepsis at the ED, 17.7–51.1% had AKI (Table 3). The highest incidence of AKI was in qSOFA positive patients with up to 51.1% according to the AKIN and KDIGO definition. Overall, patients in the qSOFA group had the highest incidence of AKI compared to the other two sepsis definitions according to all AKI definitions (qSOFA vs. SIRS and qSOFA vs. SOFA, *p* < 0.001 for all comparisons) (Table 3). In addition, patients in the SOFA group more often had AKI compared to patients with SIRS (*p* < 0.001).

**Table 3:** Incidence of AKI classified by different AKI and sepsis definitions

	AKI stage	RIFLE	AKIN	AKIB	Delta check	KDIGO	*p* Value
SIRS, *n* = 2011 (97.4% of total)	1	195 (9.7)	349 (17.4)	188 (9.3)	499 (24.8)	361 (18.0)	
	2	95 (4.7)	95 (4.7)	151 (7.5)	n.a	96 (4.8)	
	3	66 (3.3)	66 (3.3)	160 (8.0)	n.a	53 (2.6)	
	Total	356 (17.7)	510 (25.4)	499 (24.8)	499 (24.8)	510 (25.4)	< 0.001^*^
qSOFA, *n* = 315 (15.3% of total)	1	62 (19.7)	93 (29.5)	47 (14.9)	155 (49.2)	99 (31.4)	
	2	40 (12.7)	40 (12.7)	50 (15.9)	n.a	41 (13.0)	
	3	28 (8.9)	28 (8.9)	58 (18.4)	n.a	21 (6.7)	
	Total	130 (41.3)	161 (51.1)	155 (49.2)	155 (49.2)	161 (51.1)	< 0.001*
SOFA, *n* = 1246 (60.3% of total)	1	166 (13.3)	297 (23.8)	148 (11.9)	457 (36.7)	309 (24.8)	
	2	97 (7.8)	97 (7.8)	134 (10.8)	n.a	98 (7.9)	
	3	71 (5.7)	71 (5.7)	175 (14.0)	n.a	58 (4.7)	
	Total	334 (26.8)	465 (37.3)	457 (36.7)	457 (36.7)	465 (37.3)	< 0.001*
*p* Value for total AKI		< 0.001^†^	< 0.001^†^	< 0.001^†^	< 0.001^†^	< 0.001^†^	

Data are presented in absolute numbers (percentages)

*Significant differences: RIFLE vs. AKIN, RIFLE vs. AKIB, RIFLE vs. delta check, RIFLE vs. KDIGO, *p* < 0.001. With Bonferroni adjustment, a *p* value of < 0.005 was considered statistically significant

^†^Significant differences for all comparisons of total AKI: SIRS vs. qSOFA, SIRS vs. SOFA, qSOFA vs. SOFA, *p* < 0.001. With Bonferroni adjustment, a *p* value of < 0.016 was considered statistically significant

### Acute kidney injury by AKI definition

In the SIRS group, the incidence of patients with AKI ranged from 17.7% (using the RIFLE criteria) to 25.4% (according to the AKIN and KDIGO criteria) (*p* < 0.001). In the qSOFA group, the incidence of AKI was 51.1% using the AKIN and KDIGO criteria, with small differences in the severity of AKI between these two definitions.

In patients who fulfilled the SOFA criteria for sepsis, the incidence of AKI varied from 26.8% (using the RIFLE definition) to 37.3% (according to the AKIN and KDIGO criteria) (*p* < 0.001).

**Fig. 1:**
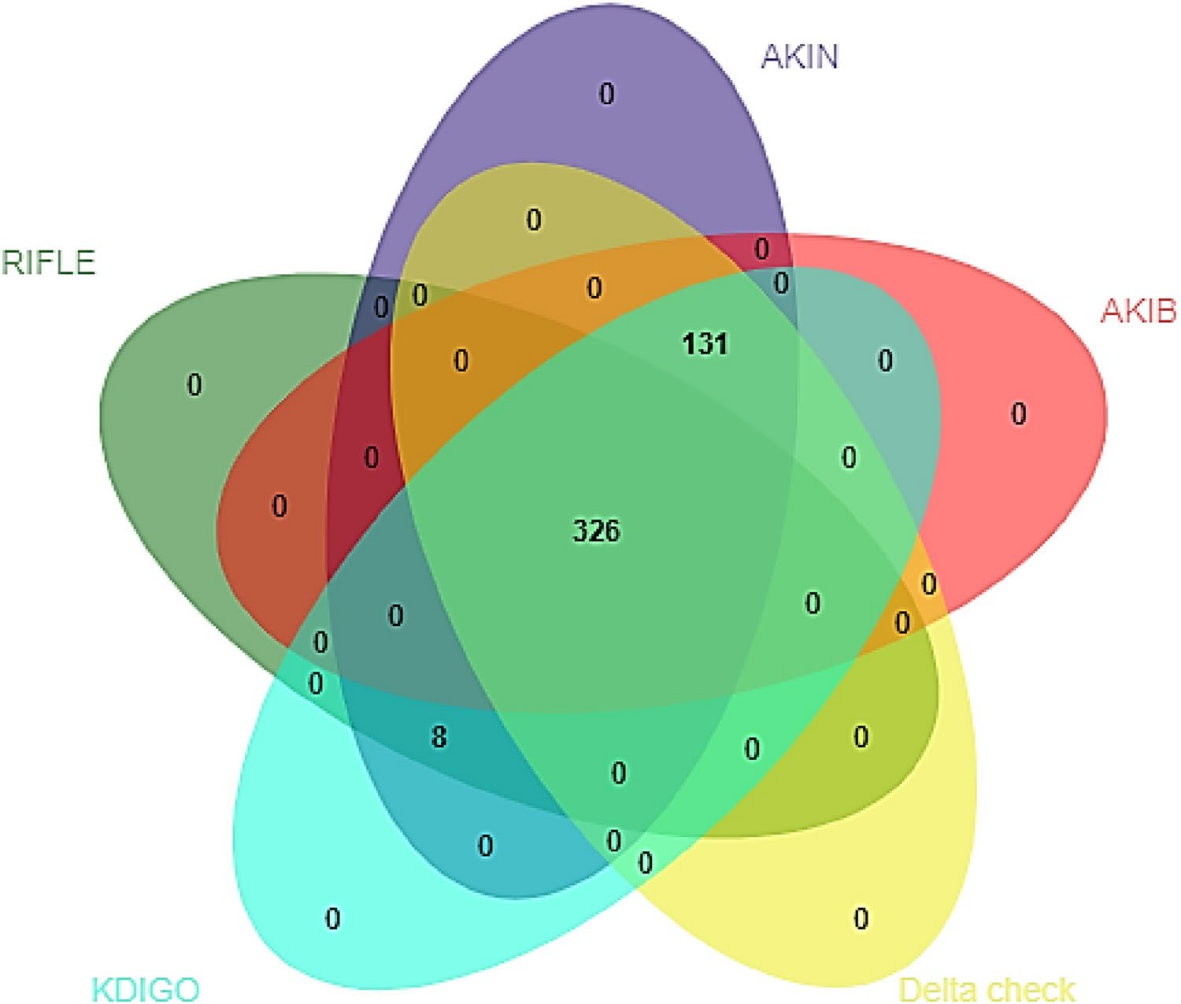
Venn diagram of overlap between different AKI definitions in patients with sepsis according to the SOFA criteria. In patients with sepsis (SOFA), all AKI definitions defined 326 patients as having AKI. All, except for the RIFLE criteria, defined another 131 patients. Another eight patients were identified by the RIFLE, AKIN, and KDIGO definition

Figure 1 shows the overlap of the total incidence of AKI for the different AKI definitions in patients with sepsis according to the SOFA criteria. A total of 326 patients were identified by all five AKI definitions. Additional 131 patients were detected by the AKIN, AKIB, delta check, and KDIGO criteria, whereas another eight patients were identified by the RIFLE, AKIN, and KDIGO criteria.

### Outcomes

In patients with sepsis according to the SOFA criteria, 5.6–7.8% of the patients with AKI had persistent kidney injury. All definitions selected the same patients (Table 4). Median length of stay was 8–9 days, irrespective of the AKI definition. Patients with positive SOFA criteria without AKI had a significant shorter length of stay (6 days (4–11), *p* < 0.001, data not shown) than those with AKI.

**Table 4:** Clinical outcomes stratified by different AKI definitions in patients with sepsis according to the SOFA criteria

	Clinical outcome	RIFLE, *n* = 334	AKIN, *n* = 465	AKIB, *n* = 457	Delta check, *n* = 457	KDIGO, *n* = 465	*p* Value
SOFA, *n* = 1246	Persistent kidney injury, relative to baseline	26 (7.8)	26 (5.6)	26 (5.7)	26 (5.7)	26 (5.6)	0.68
	Persistent kidney injury, relative to admission	3 (0.9)	3 (0.6)	3 (0.7)	3 (0.7)	3 (0.6)	0.99
	Hospital length of stay (days)	9 (5–17)	8 (5–17)	8 (5–17)	8 (5–17)	8 (5–17)	1.0
	Crude 30-day mortality	74 (22.2)	100 (21.5)	95 (20.8)	95 (20.8)	100 (21.5)	0.99

Data are presented in medians (interquartile ranges) or in absolute numbers (percentages)

In patients with AKI, 20.8–22.2% died within 30 days after admission (Table 4) [vs. 15.0% in patients without AKI, *p* = 0.23 (data not shown)]. Using the KDIGO criteria, crude 30-day mortality was higher in patients with Stage 2 and 3 AKI compared to patients with sepsis without AKI (OR 2.23, 95% CI 1.37–3.62, *p* = 0.001, and OR 2.04, 95% CI 1.10–3.80, *p* = 0.02), but not Stage 1 (*p* = 0.09) (Table 5). The same pattern was seen using the RIFLE and AKIN criteria. Using the AKIB criteria, 30-day mortality was higher in Stage 3 AKI, compared to patients with sepsis without AKI (OR 1.83, 95% CI 1.24–2.72, *p* = 0.003), but not in Stage 1 and 2 AKI (*p* = 0.33 and *p* = 0.29).

**Table 5:** Predictive ability of multivariable logistic regression models of the different AKI definitions in patients with sepsis (SOFA) with respect to crude 30-day mortality

	AKI criteria	Odds ratio (95% CI)	*p* Value
SOFA, *n* = 1246	RIFLE*		
	Risk	1.12 (0.72–1.74)	0.62
	Injury	2.02 (1.25–3.28)	0.004
	Failure	2.16 (1.25–3.74)	0.006
	AKIN*		
	Stage 1	1.30 (0.91–1.86)	0.15
	Stage 2	2.14 (1.31–3.50)	0.002
	Stage 3	2.29 (1.31–3.99)	0.003
	AKIB*		
	Stage 1	1.26 (0.79–1.99)	0.33
	Stage 2	1.29 (0.80–2.08)	0.29
	Stage 3	1.83 (1.24–2.72)	0.003
	Delta check*	1.48 (1.10–1.99)	0.01
	KDIGO*		
	Stage 1	1.35 (0.96–1.91)	0.09
	Stage 2	2.23 (1.37–3.62)	0.001
	Stage 3	2.04 (1.10–3.80)	0.02

*Patients with sepsis without AKI were used as reference

## Discussion

This multi-center study shows that the incidence of AKI in patients with sepsis in the ED varies greatly (ranging from 17.7 to 51.1%). This variation seems to be highly dependent on the definition of sepsis, but also on the definition of AKI. When AKI was present, irrespective of the definition applied, patients had a longer length of stay and high 30-day mortality.

Patients with positive qSOFA and SOFA criteria more often had AKI at presentation by any definition compared to patients meeting the SIRS criteria. Patients with positive qSOFA criteria had the highest incidence of AKI (51.5%), whereas AKI was present in almost 40% of patients with positive SOFA criteria and in up to a quarter of patients with positive SIRS criteria. The high incidence of AKI in patients with positive qSOFA criteria shows that these criteria probably select the most severely ill patients. Thus far, the qSOFA criteria are used in clinical setting as a screening instrument for sepsis. In line with these findings, we hypothesize that the qSOFA score at the ED may be interpreted as a predictor for the development of AKI.

The higher incidence of AKI in patients with positive SOFA criteria compared to SIRS may be explained by the fact that the SOFA criteria aim to select patients with more severe stages of sepsis. In addition, the SOFA score was introduced in clinical practice to indicate organ failure, including kidney failure. Serum creatinine is one of six variables of the SOFA score, which explains the association between AKI and sepsis as defined by the SOFA score.

The majority of patients had concordant AKI diagnoses, applying the five different AKI criteria, as shown by the Venn diagram. This high concordance between the AKI definitions is not remarkable, as the definitions highly overlap. However, we encountered some differences in the severity of AKI categories between the AKI definitions. In patients with sepsis according to the SOFA criteria, AKI was already present at presentation at the ED in 37.3% according to the AKIN and the KDIGO criteria, whereas the RIFLE criteria defined AKI in only 26.8% of cases (*p* < 0.001). In patients with sepsis defined by the SIRS criteria, the AKIN and KDIGO criteria defined AKI in 25.4%, compared to 17.7% when the RIFLE criteria were applied (*p* < 0.001). In patients with sepsis according to qSOFA, we retrieved similar results. The higher number of AKIN and KDIGO positive patients by any sepsis definition compared to the RIFLE criteria is mainly accounted for by the significantly higher number of stage 1 AKI. This is probably explained by the less-strict definition of stage 1 AKIN, AKIB, and KDIGO (i.e., ≥ 26.5 µmol/l increase of serum creatinine). Since the mortality numbers are comparable, the question is whether the increase in stage 1 AKI, using the AKIN and KDIGO criteria, is of clinical relevance. Furthermore, we hypothesize that the relative large proportion of patients with the most severe stage of AKI using the AKIB criteria reflects an overestimation of the most severe stage of AKI, since the ORs for 30-day mortality with this stage were substantially lower than the ORs for the most severe stages of AKI using the RIFLE, AKIN, and KDIGO criteria.

When AKI (by any definition) was already present at the ED, 30-day mortality was as high as 22.2%. The presence of AKI may therefore be seen as an early sign of more severe disease and helps to identify sepsis patients at high risk of adverse outcome. Moreover, patients with stage 2 and 3 RIFLE, AKIN, and KDIGO AKI showed similar predictions of mortality risk, whereas the AKIB and delta check criteria had lower ORs for 30-day mortality. The predictive ability of AKI (by any definition) with respect to crude 30-day mortality was insufficient in patients with positive SOFA criteria for all five AKI definitions (AUROC curves between 0.55 and 0.57, data not shown).

Other secondary outcome measures in SOFA patients such as length of stay and persistent kidney injury were not different between all five AKI definitions, whereas patients without AKI had a shorter length of stay. Persistent kidney injury was rare (26/1246, 2.1%).

Given the important clinical consequences of early recognition of AKI in sepsis, one can argue which AKI definition should be used, because the incidence of AKI varies greatly depending on the chosen AKI definition. The aim of using criteria for AKI is to recognize AKI in patients with sepsis as early as possible, because sepsis-associated AKI is related to increased mortality and morbidity, and to prevent persistent kidney injury. Therefore, the criteria should select the patients with true AKI that are at risk for persistent kidney injury and increased morbidity and mortality. In addition, they should be sensitive enough for the initial evaluation and require greater specificity for the final diagnosis. Next to this, over time, the patient’s clinical course and response to therapy can be included in the assessment of AKI. Compared to the other definitions of AKI, the KDIGO criteria identified the highest number of patients with AKI and had the highest ORs for 30-day mortality. These findings support the advice of a recent consensus statement of the 2019 KDIGO Consensus Conference, to use the KDIGO criteria to define AKI [20], including patients with sepsis.

Other studies have described the incidence of AKI in sepsis using different definitions in different settings [21]. Most of these studies were done in patients in the ICU [2, 6, 22], whereas only a few groups studied hospitalized patients in general [23, 24], or specific patient categories [25, 26], with incidences ranging from 4.8 to 87.5% depending on setting and population. None of the previous studies described the incidence of AKI in patients presenting with sepsis at the ED, nor did they use the new SOFA criteria for sepsis. In contrast to some other studies, KDIGO stage 1 AKI was not associated with an elevated risk of mortality in our study. Compared to other studies, KDIGO stage 2 and stage 3 AKI had lower ORs for 30-day mortality than previously reported [22, 27]. This finding may be explained by the fact that we included ED patients, who represent the whole spectrum of sepsis severity, including patients in an early and probably reversible stage of the sepsis syndrome, whereas the above-mentioned studies included ICU patients only.

Our multi-center studied a large population of patients with the whole spectrum of sepsis which is a good reflection of the daily practice in the ED. However, there are some limitations. Our study focused on the incidence of AKI already present at presentation at the ED. Some patients develop AKI later on during their clinical course. In addition, our study did not include urine production. Urine output may be a sensitive indicator of acute kidney injury [3]. However, standardized documentation of urine production is a difficult parameter to retrieve in retrospective studies, especially in the ED. Finally, back-calculating missing baseline serum creatinine values may result into under- or overestimation of AKI [10]. The number of patients with a missing baseline serum creatinine in our study was low (2.6%). In contrast to other reports, we used the lowest serum creatinine during 3 month follow-up as baseline, instead of the lowest creatinine value during an admission [16]. This way, the renal function was allowed to recover even more, compared to a time frame of only the length of admission. We believe that it is therefore a more accurate way of back-calculating baseline serum creatinine. Because of this and because of the low proportion of patients with a back-calculated baseline creatinine, we think that potential under- or overestimation of AKI is negligible in our study.

In conclusion, this is the first study comparing different AKI definitions and applying different sepsis definitions, including the new SOFA criteria for sepsis. Our multi-center study in a large population of ED patients with sepsis shows that the incidence of AKI in patients with sepsis is highly dependent on how patients with sepsis are categorised and how AKI is defined and ranges from 17.7%—51.1%. In patients with sepsis meeting the SOFA criteria, almost 40% of patients were classified as having AKI at the ED. When AKI is already present at the ED, this is associated with a longer length of hospital stay and a high 30-day mortality rate (up to 22.2%, especially when AKI is more severe), irrespective of which AKI definition is used. Early recognition of sepsis-associated AKI in patients in the ED is important because of the more severe prognosis. Therefore, knowledge of the different AKI definitions is mandatory. This study provides knowledge on the use of the different AKI and sepsis definitions.

## Data Availability

The datasets that support the findings of the current study are available from the corresponding author on reasonable request.
